# Marrying up new trends in clinical research: AI and -omics

**DOI:** 10.1192/j.eurpsy.2025.256

**Published:** 2025-08-26

**Authors:** D. Cavaleri

**Affiliations:** School of Medicine and Surgery, University of Milano-Bicocca, Monza, Italy

## Abstract

**Abstract:**

Artificial intelligence (AI) and -omics techniques (genomics, proteomics, metabolomics) represent two rapidly evolving fields that are increasingly intersecting to transform clinical research and healthcare.

AI, aiming to mimic human intelligence through computational modelling, possesses extraordinary capabilities for big data analysis. -Omics, offering quantifiable and dynamic readouts of the molecular state of the subject, can generate large databases covering hundreds to thousands of molecules with complex relationships.

Combining AI-driven insights with the wealth of data generated by genomics, transcriptomics, proteomics, and metabolomics can help uncovering complex biological networks, with the potential to revolutionize our understanding of disease mechanisms, improve patient stratification, and optimize therapeutic interventions.

In this presentation, the concepts of AI and -omics and their combined application to clinical research will be discussed, summarizing the strengths and limitations of these approaches. Studies leveraging AI across various -omics domains will be presented. Key advances, ongoing challenges, and future perspectives in this rapidly evolving field will be debated.

While still in the early stages, the synergistic partnership between metabolomics and AI represents an exciting frontier that holds great promises for groundbreaking advancements in clinical research and human health, paving the way toward a new era of precision medicine.

**Disclosure of Interest:**

None Declared

